# *GBA*, Gaucher Disease, and Parkinson’s Disease: From Genetic to Clinic to New Therapeutic Approaches

**DOI:** 10.3390/cells8040364

**Published:** 2019-04-19

**Authors:** Giulietta M. Riboldi, Alessio B. Di Fonzo

**Affiliations:** 1 The Marlene and Paolo Fresco Institute for Parkinson’s and Movement Disorders, NYU Langone Health, New York, NY 10017, USA; 2 Dino Ferrari Center, Neuroscience Section, Department of Pathophysiology and Transplantation, University of Milan, 20122 Milan, Italy; alessio.difonzo@policlinico.mi.it; 3 Foundation IRCCS Ca’ Granda Ospedale Maggiore Policlinico, Neurology Unit, 20122 Milan, Italy

**Keywords:** glucocerebrosidase, Parkinson’s disease, Gaucher’s disease, Lewy Body Dementia, REM sleep behavior disorders

## Abstract

Parkinson’s disease (PD) is the second most common degenerative disorder. Although the disease was described more than 200 years ago, its pathogenetic mechanisms have not yet been fully described. In recent years, the discovery of the association between mutations of the *GBA* gene (encoding for the lysosomal enzyme glucocerebrosidase) and PD facilitated a better understating of this disorder. *GBA* mutations are the most common genetic risk factor of the disease. However, mutations of this gene can be found in different phenotypes, such as Gaucher’s disease (GD), PD, dementia with Lewy bodies (DLB) and rapid eye movements (REM) sleep behavior disorders (RBDs). Understanding the pathogenic role of this mutation and its different manifestations is crucial for geneticists and scientists to guide their research and to select proper cohorts of patients. Moreover, knowing the implications of the *GBA* mutation in the context of PD and the other associated phenotypes is also important for clinicians to properly counsel their patients and to implement their care. With the present review we aim to describe the genetic, clinical, and therapeutic features related to the mutation of the *GBA* gene.

## 1. Introduction

*GBA* is a gene located on chromosome 1 (1q21) encoding for the glucocerebrosidase (GCase), a lysosomal enzyme involved in the metabolism of glucosylceramide. The mutation of this gene has been classically associated with Gaucher’s disease, a systemic disorder with a variable degree of involvement of the central nervous system. Surprisingly, about 14 years ago it was observed that mutations in this same gene were associated with an increased incidence of Parkinson’s disease (PD), in both Gaucher’s patients as well as asymptomatic carriers [1,2,3,4]. PD is the second most common neurodegenerative disorder, affecting 2–3% of the world population over the age of 65 [5]. It is caused by the progressive loss of dopaminergic neurons in the substantia nigra. Classically it presents with a combination of bradykinesia, rigidity, resting tremor, and postural instability. However, a list of non-motor features, such as hyposmia, constipation, urinary symptoms, orthostatic hypotension, anxiety, depression, impaired sleep, and cognitive impairment can present as well in various degrees [5]. Since the first observations of *GBA* and PD, their association has been extensively explored. Different hypotheses have been formulated to explain the causative role of this mutation in PD [6]. First of all, GCase is part of the endolysosomal pathway, which seems to be particularly crucial in the pathogenesis of PD. Indeed, many different monogenic familial forms of PD are caused by genes involved in this pathway [7]. Moreover, mutated GCase is not able to fold properly and thus can accumulate in different cellular compartments of the dopaminergic neurons, causing a cell stress response that can be deleterious of the cells. In addition, impaired GCase activity seems to cause an accumulation of alpha-synuclein (for a comprehensive review see [8]).

Today we know that *GBA* mutations are the major genetic risk factor for PD. Impaired GCase activity has been identified also in idiopathic cases of PD patients who did not carry a mutation in the gene, suggesting a central role of this enzyme in the pathogenesis of the disease [9,10].

In the present review, we aim to summarize the genetic changes and the characteristic features associated with the mutations of this gene, spanning from Gaucher’s disease to PD and the other described phenotypes. This will aid in a better understanding of the pathogenic role of this mutation. The identification of these phenotypes will allow for clinicians to offer more appropriate counseling to the patients and their families.

## 2. Pathogenetic Mutations of the *GBA* Gene

### 2.1. GBA Mutation and Gaucher’s Disease (GD)

Gaucher’s disease (GD) is a systemic disorder that can present with a various degree of systemic and neurological manifestations. According to the severity of the disease and the neurological involvement, three different types of GD have been identified. GD type 1 has been classically considered only a systemic disorder, with no neurological involvement whatsoever. Anemia, leukopenia, thrombocytopenia with frequent bleeding, osteopenia with bone pain, easy fractures, Erlenmeyer flask deformity, as well as hepatosplenomegaly, failure to grow, and puberty delay can be presenting features of this disease [11,12,13,14]. Monoclonal gammopathy has been reported as well [15]. The disease can manifest early in childhood but it may remain undiagnosed until adulthood when the phenotype is mild. The pathological hallmarks of the disease are the so-called Gaucher cells, macrophages engorged with aberrant lysosomes as a consequence of the GCase-impaired activity. Symptoms are caused by the infiltration of these cells in the reticuloendothelial system of the affected organs [16]. In recent years, the natural history of GD type 1 has dramatically changed since the introduction of target treatments, such as enzyme replacement therapy (ERT) (human recombinant enzyme to be administered intravenously every other week) and oral substrate reduction therapy (SRT) [17]. Treatments with these two approaches are able to address the majority of the systemic symptoms associated with GD type 1 and those in GD type 3. So far, SRT has been approved only for subjects over the age of 18 years. However, in the adult population it represents an important alternative first line treatment. Unfortunately, these therapies are not able to cross the blood-brain barrier and therefore they are not suitable for the treatment of the neurological complications associated with GD type 2 and 3. The two latest forms are also referred to as the acute (type 2) and chronic (type 3) neuronopathic form. Patients affected with GD type 2 start manifesting severe symptoms very early, usually within the first six months of life. They usually present a combination of severe neurological manifestations, with brainstem involvement (i.e., eye movement abnormalities, spasticity, hypotonia) and seizure, as well as life-threatening systemic symptoms, such as respiratory distress and aspiration pneumonia [18,19]. Skin manifestations, like ichthyosis or collodion abnormalities, as well as hydrops fetalis, can be present. Prognosis is very poor and death usually occurs before the age of four [20]. GD type 3 (chronic neuronopathic form) has been further classified as GD type 3a,b,c. GD type 3a presents a milder visceral phenotype, but can be associated with severe and life-threatening myoclonic seizures. GD type 3b, instead, is characterized by a more prominent visceral involvement [21]. Interestingly, one of the features that have been used to try to discriminate between patients with GD type 1 and the milder neuropathic form GD type 3 is the assessment of the eye movements. Indeed, patients with GD type 3, especially type b, present with characteristic eye movement abnormalities. In particular they show loss of horizontal before vertical gaze palsy and slowing of the saccades, suggesting involvement of the brainstem. GD type 3c, instead, is the only subtype of the disease presenting with cardiac mitral and aortic calcification and poor prognosis [21]. A particular cluster of patients with GD type 3 has been identified among the Swedish population. This is also referred as Norrbottnian form, because of its geographical distribution. It is associated with the c.1448T > G mutation and it presents with an early and severe splenomegaly and a combination in the first or second decade of ataxia, spastic paresis, horizontal supranuclear gaze palsy, kyphoscoliosis and other orthopedic abnormalities, cognitive impairment, and seizures [22].

Those different phenotypes are associated with discrete genetic mutations, as detailed below.

#### Different Pathogenic Mutations of *GBA* Associated with Gaucher’s Disease (GD) Subtypes

**Figure 1:**
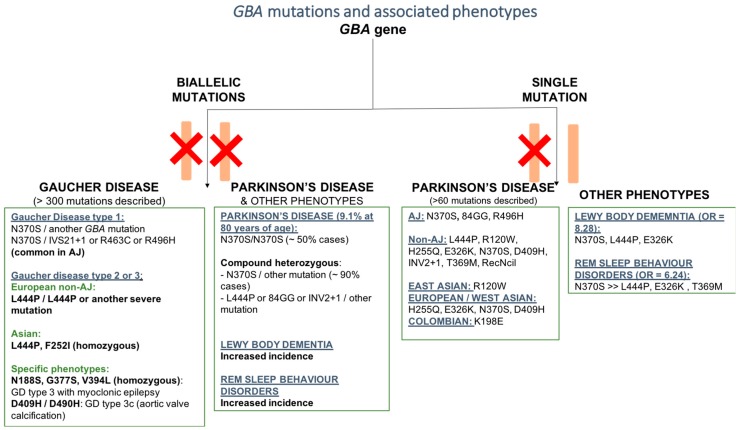
Schematic representation of the most common pathogenic mutations of the *GBA* genes and associated phenotypes. Phenotypes were grouped based on homozygous and heterozygous mutations, ancestry, and specific associated features.

More than 300 variants of the *GBA* gene have been associated with Gaucher’s disease [23]. GD is an autosomal recessive disorder. In order for the disease to manifest, patients need to carry a pathogenic mutation on both alleles of the GBA gene, either in a homozygous or compound heterozygous fashion. Point mutations, insertion, deletion, missense mutations, splice junctions, and concomitant multiple mutations have been reported [24]. The different variants can be more represented in particular ethnic groups as well as in particular phenotypes. The c.1226A < G (N370S; or N409S according to the new nomenclature) mutation is the most common one among Ashkenazi Jew (AJ) patients, followed by the c.84dupG (84GG) mutation, which is more rare. The c.115 + 1G > A (IVS2 + 1), c.1504C > T (R463C), and c.1604G > A (R496H) are commonly found in AJ patients with GD type 1 [24]. On the contrary, the N370S mutation is rarely found among Chinese and Japanese patients [24] (Hruska et al., 2008). Among Asian ethnic groups, the c.1448T > C (L444P, or L483P according to the new nomenclature) and the c.754T > A (F252I), usually associated with GD type 2 and 3, are more prevalent, also explaining why among these populations the neuropathic forms of GD are more frequent [20]. c.1448T > C (L444P) is also the most frequent mutation among Caucasians with a non-Ashkenazi Jew ancestry [25] (Figure 1).

Different mutations can lead to different phenotypes of GD. The c.1226A > G (N370S) mutation is associated only with Gaucher’s disease type 1 and it seems to be protective for the development of the neurological involvement characteristic of GD type 2 and 3. Indeed, patients who present the c.1226A > G (N370S) mutation on at least one allele of the *GBA* gene will manifest only GD type 1 [24]. Interestingly, subjects who are homozygous for the N370S variant can also remain asymptomatic for the disease. On the other hand, the c.1448T > C (L444P) mutation is usually associated with GD type 2 or 3, even when presenting in a compound heterozygous state [19]. Homozygous c.1448T > C (L444P) mutation [c.1448T > C]1[c.1448T > C] (L444P/L444P) with no recombinant alleles can be associated with very severe but also milder phenotypes [26]. The c.1342G > C (D409H) variant is responsible for GD type 3c which presents with characteristic cardiac valve calcifications [27]. c.680A > G (N188S), c.1246G > A (G377S), and c.1297G > T (V394L) are more likely to be associated with myoclonic epilepsy [28,29,30]. Despite previously reported observations, it is commonly found that members of the same family report variability in the manifestation of symptoms even with an identical genotype, suggesting that a genotype/phenotype correlation is tentative still. Other reported mutations are uniquely rare and oftentimes private among specific families. [12]. Hence, it is difficult to make generalizations about these mutations specific phenotypical profiles.

Another interesting mutation is the c.1093G > A (E326K), which caused a lot of debate in the literature [31]. Indeed, it is not clear whether this mutation is really pathogenic for GD, since it was found also in a significant number of asymptomatic carriers in homozygosity [32,33]. However, when associated with other *GBA* mutations on the same allele, it can cause severe impairment of the GCase activity [34,35]. Interestingly enough, the same mutation seems to be significantly associated with an increased risk of PD [33].

### 2.2. GBA Mutation and Parkinson’s Disease (PD)

#### 2.2.1. Pathogenic Mutations of *GBA* Associated with PD

More than a decade ago, the association between an increased risk of developing PD and the presence of *GBA* mutations was initially noticed in large Gaucher’s disease clinics. The incidence of PD among GD patients and their relatives, which were supposedly carriers for the mutation, seemed to be higher than the general population. Initially, only single case reports were suggesting this association. Interestingly, PD was noticed in patients with GD type 1, which has always been considered the non-neuropathic form of the disease [36,37,38,39,40,41]. It was only when larger populations of PD patients were screened for mutations of this gene that the important role of *GBA* in the pathogenesis of PD was assessed worldwide.

So far, more than 50 population studies have screened the *GBA* gene among PD patients, covering a large number of ancestries (reviewed in [42,43]). Overall, these studies demonstrated that the incidence of *GBA* mutations is significantly higher among PD patients, compared to non-affected subjects. Compared to GD, a smaller number of *GBA* mutations have been reported in patients with PD (about 130 *GBA* mutations) [42]. However, in many of these studies, only the mutations that are most commonly associated with PD were screened. Therefore, less frequent variants still associated with the disease could go undetected. Among all, the c.1226A > G (N370S) and the c.1448T > C (L444P) mutations are the two most common mutations worldwide. Indeed, in some populations they account for the 70–80% of the total number of variants of *GBA* associated with PD [44]. Among subjects from eastern Europe with an AJ ancestry, the c.1226A > G (N370S) mutation is definitely the most frequent one among PD patients, as already reported for GD (Figure 1). Among the non-AJ European descendants, the c.1448T > C (L444P) mutation is more common. Interestingly, it has been reported that some mutations are able to increase the risk of PD only in the context of specific ancestry [42]. This is the case of the c.84dupG (84GG) and c.1604G > A (R496H) for AJ subjects, the c. 475 C > T (R120W) for East Asian populations, and the c.882T > G (H255Q), c.1093G > A (E326K), c.1342G > C (D409H), and c.1226A < G (N370S), which are only found in subjects of European or West Asian ancestry [42] (Figure 1). A recent study identified an increased incidence of the K198E variant (previously described in GD1 and GD2 patients) in a population of PD patients from Columbia compared to controls [43]. It seems that severe *GBA* mutations (as classified according to the subtype of GD that they are associated with), such as c.84dupG (84GG), c.115 + 1G >A (IVS2 + 1), c.1297G > T (V394L), c.1342G > C (D409H), c.1448T > C (L444P), and c.1263del + RecTL, are associated with a higher risk of causing PD compared to milder mutations, such as the N370S and c.84dupG (84GG) [45]. Moreover, severe mutations are associated with an earlier age of onset, as well as a more rapid progression and increased involvement of cognitive functions [45,46,47]. In one study, the motor and some of the non-motor symptoms (such as depression, REM sleep behavior disorders, and hyposmia) were significantly worse in PD patients carrying severe *GBA* mutations compared to subjects carrying mild mutations or with idiopathic PD [48].

Interestingly, *GBA* represents only a risk factor for PD. This means that not every carrier will develop the disease. The reason for the reduced penetrance of these mutations has not yet been fully elucidated. Based on large population studies, today we know that, among *GBA* carriers, about 9.1% will develop PD. Some reports suggest that the penetrance of PD in GD patient is 30% at 80 years, but this data needs to be confirmed by further studies [49]. Patients with a homozygous mutation of *GBA*, thus affected with Gaucher’s disease, have a higher risk of developing PD and usually with an earlier age of onset of symptoms [48]. Having said that, it is worth noticing that the majority of subjects with GD will never develop PD, even in the case of severe mutations. It is still controversial whether PD in patients with GD presents with a more severe phenotype compared to *GBA* carriers. Carriers of the *GBA* mutation harbor an increased risk of developing PD by five times in heterozygous carriers and 10–20 times in homozygous carriers [50,51,52,53]. *GBA* mutations are present in about 2–30% of PD patients [54]. Carrier frequency can be very different across different ancestry. Among AJs, it goes from 10 to 31%, while in Norwegian’s it is only 2.3% [54]. In patients of European non-AJ ancestry, it ranges from 2.9 to 12% [54].

In the last few years, there has been a great effort to try to clarify the pathogenic role of the *GBA* mutations in PD and many different hypotheses have been formulated, as reported above (for review see [8]). It is important to note that a growing amount of data is suggesting a failure of the endolysosomal and of the autophagic pathways in PD [55]. These scavenger systems are crucial for the degradation of alpha-synuclein, whose accumulation in the dopaminergic neurons is one of the hallmarks of PD. In the lysosome, GCase plays an important contribution for these processes and, in particular, in the interplay with alpha-synuclein [56]. Therefore, it is not totally surprising that a dysfunction of this enzyme is related to PD. How the different mutations of *GBA* that have been described in PD patients are able to affect the activity of the GCase has not been fully understood. We know that the GCase has three active domains. PD-associated mutations are found in distinctive domains of the protein. The c.1342G > C (D409H) and c.1297G > T (V394L) variants are located in domain I. The c.84dupG (84GG) mutation causes a frameshift that can induce aberrantly shorter or longer proteins that are non-functioning [23]. Other mutations, instead, are not found in the functional domains but do interfere with the final structure of the enzyme, thus making it more unstable or affecting its interaction with other proteins. The c.1226A > G (N370S) and c.1448T > C (L444P) mutations are, for example, located in the proximity of the binding site of the Saposin C, an activator of GCase [57]. More importantly, SapC competes with the binding of alpha-synuclein to GCase, which would cause the inhibition of the enzyme [58,59]. Interestingly, the c.1226A > G (N370S) mutation also seems to affect the ability of the GCase to modify the conformation of one of its loops, loop 3, according to changes in pH [60,61]. Conformational changes in response to the changes of the cellular environment are critical for the proper function of the protein. Despite our knowledge about the structural effects of the different mutations, the exact correlation between the localizations of pathogenetic variants of the gene and the degree of expression of PD has not yet been fully described.

It is also worth noting that *GBA* presents a pseudogene (*GBAP1*) that shares a very high degree of homology—96% sequence identity–located in the proximity of the original gene [62,63]. Therefore, genetic analysis will have to take this into account and should be performed in a specialized laboratory in order to obtain reliable results. New technologies, such as the long-read sequencer, are on the horizon for even more in-depth identification of possible *GBA* mutations [64].

#### 2.2.2. *GBA* Mutations and Parkinson’s Disease Phenotype

PD patients carrying *GBA* mutations are not easily recognizable in most cases because they do not present exclusive features that would clearly distinguish them from patients with idiopathic PD (iPD). However, large population studies comparing carriers vs. non-carriers, mild vs. severe mutations, as well as heterozygous manifesting carriers vs. PD–GD patients, allowed the ability to define common traits in these subgroups of patients (for a comprehensive review see [8]). In particular, *GBA*–PD patients present an overall earlier age of onset compared to non-carriers. Disease manifests about 3–6 years earlier in heterozygous carriers, irrespectively of the severity of the mutation, and about 6–11 years earlier in subjects with homozygous mutations [45,46,48,54,65,66,67,68,69,70]. There are limited reports of *GBA* mutation carriers having an age of onset in the 20′s. [31,54,71,72,73].

The progression of the disease has been characterized in many different studies by a more pronounced cognitive deficit in a significant percentage of these patients, with a risk of developing dementia up to three times higher compared to iPD, which is even more increased in patients with severe mutations [46,48,74]. Hallucinations and REM sleep behavior disorders (RBD) also are more common among *GBA* patients in a dose-dependent fashion, being more frequent in subjects with homozygous mutations and in patients carrying severe vs. milder mutations. However, other non-motor symptoms, such as depression and anxiety, constipation, urinary symptoms, orthostatic hypotension, and sexual dysfunctions are over-represented as well in *GBA* carriers compared to iPD, especially in the presence of severe mutations, but with no increased severity in GD patients [46,48,75,76]. An increased incidence of dysautonomic features has been suggested to be the main driver of the slightly reduced survival reported in these patients [77]. Motor complications, such as dysphagia, dysarthria, and freezing of gait, are more frequent as well in *GBA* carriers [46,67].

In patients with *GBA* mutations and PD, the rigid akinetic phenotype seems to be more common. Usually, these patients present a very good response to levodopa, although the progression of the motor symptoms can be slightly faster compared to iPD but without higher rates of motor fluctuations or dyskinesia. Therefore, no specific treatment approaches need to be considered for this subgroup of patients. Interestingly, a recent study evaluated the outcomes of treatment with deep brain stimulation (DBS) in a cohort of PD patients carrying *GBA* mutations [78]. After a follow up of 7.5 years on average, it was noticed that the het-*GBA* cohort presented similar outcomes compared to iPD in terms of motor symptoms, while cognitive impairment and non-motor symptoms were definitely more represented among carriers [78]. However, because of the beneficial effect on the motor symptoms, DBS should be considered as a suitable option for these patients.

#### 2.2.3. *GBA* Mutations and Other Phenotypes

*GBA* mutations were identified also in cases of REM sleep behavior disorders (RBD) and in cases of dementia with Lewy bodies (DLB) [79].

##### *GBA* and Dementia with Lewy Bodies

A relatively low number of studies have been conducted to explore the incidence of the *GBA* mutation among patients affected with dementia with Lewy bodies (DLB), which was found to be even higher compared to the one in PD patients. In a cohort study of DLB patients, the frequency of *GBA* mutations was 7.49% with an odd ratio of 8.28 [79]. In another study in Spanish subjects, and in a number of autoptic brain tissues from pathologically proven DLB patients, a *GBA* mutation was identified in 12–13% of the cases [80]. Recent genome-wide association studies (GWAS) also confirmed the significant association between *GBA* mutations and DLB (particularly the rs35749011 variant) [81]. Among *GBA* carriers, the risk of developing DLB is about three times greater than developing PD [82].

As well as in PD patients, *GBA* mutations are associated with an earlier age of onset in DLB cases compared to non-carriers (of approximately five years) and a higher disease severity score [79,80]. The association between *GBA* mutations and DLB was found to be higher among male subjects compared to female [80]. These observations were confirmed also in a following study in a cohort of patients with DLB and AJ ancestry [83]. *GBA* mutation carriers (about 11% of the entire cohort) presented more severe symptoms, particularly in terms of increased hallucinations, worse RBD symptoms, and overall cognitive and motor features [83].

A number of different mutations of the *GBA* gene have been reported in DLB patients. Other than the two mutations most frequently associated with PD (c.1226A > G (N370S) and c.1448T > C (L444P)), the E326K variant is over-represented in this cohort of patients compared to controls [79,80]. Interestingly, the c.1093G > A (E326K) mutation also is frequently found in patients with PD dementia (PDD) [84].

Neuropathological data does not significantly differ between DLB patients with and without a *GBA* mutation [79]. However, *GBA* carriers present a reduced GCase activity as well as a more pronounced alteration of lipid profiles in the brain [85]. *GBA* expression profiles have been shown to be reduced in DLB and PDD cases in both specific brain regions (temporal cortex and caudate nucleus respectively) and in the peripheral blood [86]. *GBA* mutations are more significantly associated with Lewy bodies (LB) pathology (especially with a cortical localization) than with Alzheimer’s disease (AD) pathology (i.e., beta-amyloid and neurofibrillary tangles inclusions) [87].

##### *GBA* and REM Sleep Behavior Disorders

REM sleep behavior disorders (RBDs) are considered one of the prodromal symptoms of PD and patients affected by this disorder may present with alpha-synuclein accumulation in the brain [88]. According to a recent metanalysis, patients affected with RBDs present an estimated risk of developing a neurodegenerative disorder up to 97% after more than 14 years of follow up [89]. The majority of the cases who present a phenoconversion will develop an alpha-synucleinopathy, represented by PD in the majority of the cases, but also Multiple System Atrophy (MSA), Dementia with Lewy Bodies (DLB), and PD with dementia [90]. In fact, subjects with RBD may present clinical symptoms fulfilling the criteria for prodromal PD in up to 74% of the cases, manifesting worse performances in both motor and non-motor assessments compared to non-affected subjects [91,92]. Notably, many of the studies in this field did not take into consideration the significance of a family history of a neurodegenerative disorder, therefore, it is probable that the percentage of patients that reported a neurodegenerative disease is misrepresented. It would be worth exploring this aspect in future studies.

RBD seems to be more frequent in PD patients with *GBA* mutations compared with patients without this mutation (OR 3.13) [48,65,67,76]. RBDs are also more frequent in PD patients with concomitant GD than in heterozygous carriers [48]. Based on these observations, a few studies explored the incidence of the *GBA* mutation among patients affected with RBD [65,91,92,93]. These studies reported that among patients with idiopathic RBDs there is an increased frequency of *GBA* mutations (2.6–11.6% of RBD patients vs. 0.4–1.8% of the controls) [65,91,93]. A number of different *GBA* mutations were identified in RBD patients [65,93]. Some of these mutations have already been reported in PD patients, while others still do not have a clear pathogenic role. Among all the reported mutations, the two more commonly found in PD (i.e., c.1226A > G (N370S) and c.1448T > C (L444P), with N370S >> L444P), together with the c.1093G > A (E326K) and the c.1223C > T (T369M), were the most frequently represented in subjects with RBD [65,91,92,93].

Subjects with homozygous *GBA* mutations, thus affected with GD, and heterozygous carriers with no PD, presented significant worsening of rapid eye movement sleep behavior disorder scores over a period of time of two years compared with non-carrier subjects [92]. Among *GBA* carriers, the odds ratio (OR) for RBD was 6.24 (95% CI 3.76–10.35, *P* < 0.0001) [65]. The presence of *GBA* mutations does not seem to increase the risk among RBD patients of phenoconverting into PD [93]. These observations all together suggest that *GBA* may play a role in the development of RBDs, but not necessarily in determining more severe phenotypes.

Interestingly, no mutations of the *LRRK2* gene, the other common genetic risk factor for PD, have been identified so far in patients with RBDs [91,94].

## 3. New Targeted Treatments for *GBA*–PD Patients

Despite the very successful treatments that are now available to address the systemic manifestations of Gaucher’s disease, unfortunately these approaches (i.e., enzyme replacement therapy and substrate reduction therapy) are not able to reach the central nervous system and thus fail to address the neurological symptoms caused by the disease. Different companies have been working for years to try to address this issue, producing very promising results in cellular and animal models. We are now in a very exciting era where some of these experimental approaches are starting to reach the clinical scene. The treatments available so far in clinical trials try to address two main mechanisms that are thought to be detrimental in linking *GBA* mutations to PD. The first hypothesis is that mutated forms of *GBA* are not able to fold properly in the endoplasmic reticulum (ER) in the cells, causing the protein to accumulate in this cellular compartment [95]. This would trigger a stress response in the dopaminergic neurons leading to their damage and death [95]. Also, the entrapment of the beta GCase in the ER causes reduced levels of the enzyme in the cells, triggering alpha-synuclein accumulation [95]. In order to target this pathogenic mechanism, different chaperones, which are proteins able to facilitate the refolding of their substrates, were tested [95,96,97,98,99]. In 2016, a clinical study assessing the efficacy of ambroxol, one of these chaperones that showed very exciting preliminary results, was started (NCT02914366 study: https://www.clinicaltrials.gov/ct2/show/NCT02914366?cond=gba+parkinson&rank=7). This is a phase 2 clinical trial to assess the safety and the efficacy of this drug to improve motor and cognitive features of PD patients with a *GBA* mutation. The study is currently ongoing. Another similar approach has been tested in a phase 1 study by Allergan with LTI-291, a chaperone molecule able to increase the activity of GCase (https://lti-staging.squarespace.com/our-science/#lti-291). Isofagomine is another chaperone protein that has been tested in vitro and in vivo to assess its ability to modulate the phenotype induced by mutations of *GBA* [97]. This molecule is an inhibitory chaperone whose role would be the stabilization of the GCase. Clinical trials with this molecule are not available at the moment. It is also worth considering that small molecules, such as chaperones, can present different therapeutic profiles in carriers of the different mutations of *GBA* according to the effect of these variants on the protein [100].

The second mechanism that has been explored to treat *GBA*–PD patients is the accumulation in the dopaminergic neurons of glucosylceramide (the substrate normally degraded by the GCase) because of the mutation of *GBA* [101,102,103]. Genzyme recently started a multicenter, randomized, double-blind, placebo-controlled phase 2 study to assess the safety, pharmacokinetics, and pharmacodynamics of an oral compound, ibiglustat (GZ/SAR402671), which is able to reduce the levels of beta-glucocerebrosidase in *GBA* carriers with early-stage PD (MOVES-PD study: https://www.clinicaltrials.gov/ct2/show/NCT02906020?cond=gba+parkinson&rank=2). It is still a long road for the establishment of an effective treatment, but many paths have been established, giving hope for patients with PD.

Mutated GCase is more unstable compared to the wild-type form. Therefore, modulation of the degradation of GCase could be another suitable strategy to increase the activity of the enzyme and thus tackle alpha-synuclein accumulation and neurodegeneration. Hsp90β, together with other heat shock proteins (HSP), such as Hsp27, parkin, and the endoplasmic reticulum-associated pathway, are responsible for the degradation of misfolded GCase. In particular, histone deacetylase inhibitors (HDACis) and direct inhibitors of specific HSP are able to increase the GCase activity, reducing its degradation [104]. Indeed, HDACis prevent the interaction between Hdp90β and GCase through the hyperactivation of one of its domains [105].

GCase plays an important role in the autophagy-lysosomal pathway (ALP), where other genes that have been associated with PD, such as *ATP13A2,* scavenger receptor class B member 2 (*SCARB2*), sphingomyelin phosphodiesterase 1 (*SMPD1*), and others, are also involved (Moors et al., 2016). Failure of the ALP seems to be responsible for the accumulation of alpha-synuclein in neurons. Therefore, a number of pharmacological approaches directed to the ALP have been attempted in cellular and animal models of PD (for a comprehensive review see [106]). However, the autophagic pathway is broadly represented and active in different cell types and tissues in the organism. Therefore, the identification of approaches with a high selectivity for certain tissues (such as the dopaminergic neurons) or for specific mechanisms within ALP (such as GCase failure) is detrimental for the achievement of effective but also safe treatments for patients.

In order to restore GCase activity, whose failure seems to be responsible for its neuronal pathogenicity, gene therapy approaches are also in the pipeline. Preclinical studies showed that delivery of *GBA* using adeno-associated virus 1 (AAV1) in A53T–alpha-synuclein mice is able to reduce alpha-synuclein accumulation in the brain [107,108]. The field of gene therapy is now continuously growing in the context of the neurodegenerative disorders [109]. Clinical trials to assess the efficacy of this type of approach may soon be a reality in the context of PD and *GBA* mutations.

## 4. Conclusions

The discovery of the association between mutations of the *GBA* gene and PD allowed important considerations and discoveries that are contributing to a better understating of the pathogenesis of PD. Indeed, after this initial observation, the role of lysosomal impairment has been extensively explored in PD. A growing amount of emerging evidence supports the idea that the endolysosomal trafficking is involved in alpha-synuclein accumulation and dopaminergic neuron degeneration. A number of genes involved in monogenic forms of PD or genetic risk factors for the disease (such as *SNCA*, *ATP13A2*, *VPS35*, *DNAJC6*, *SYNJ1*, *LRRK2*, *RAB39B*) are part of this pathway (for review see [110]). Mutations of genes involved in the endolysosomal pathways are responsible for a group of disorders designated as Lysosomal Storage Disorders (LSD). These are typically rare autosomal recessive diseases which cause systemic involvements with variable degrees of severity and neurological involvement, usually presenting during childhood (reviewed in [111]). It is interesting to note that an increased burden of LSD-associated mutations has been identified in the screening of large PD populations compared to controls [112]. At the same time, among the 39 new gene loci associated with PD reported in the largest genome wide association study (GWAS) performed in PD patients so far, a number of these variants were found in LSD-associated genes (i.e., *NAGLU*, *GUSB*, *NEU1*, and *GRN*) [113].

The case of autosomal recessive conditions causing severe and rare disorders during childhood, which in turn present as genetic risk factors for common adult neurodegenerative disorders when in a heterozygous state, appears to be more and more frequent, usually presenting an incomplete penetrance. This is the case for a number of LSD in the context of PD or of a parkinsonian degeneration, such as *SMPD1* (sphingomyelin phosphodiesterase, Niemann–Pick disease), *ATP13A2* (P5-type ATPase, Kufor–Rakeb disease), *GALC* (galactosylceramidase, Krabbe disease), *NPC1* (Niemann–Pick type C), *NAGLU* (α-N-acetylglucosminidase, Sanfilippo syndrome B, or mucopoly-saccharidosis III disease B (MPS-IIIB)), *HEXB* (β-hexosaminidase B, Sandhoff disease (GM2 gangliosidosis)) (summarized in [114]). The association between *GBA* mutations, GD, and PD must be just the tip of the iceberg of a larger phenomenon, where the association between genes initially considered responsible only for autosomal recessive disorders turned out to be risk factors for common neurodegenerative conditions. This association may have been recognized first in GD patients because of the higher frequency of this disease compared with other LSD.

Interestingly, this is also the case for the *TREM2* gene (encoding for Triggering Receptor Expressed on Myeloid cells 2), which seems to be the most frequent genetic risk factor of another common neurodegenerative disorder, Alzheimer’s disease (AD) [115]. Autosomal recessive mutations of *TREM2* are responsible for the rare, juvenile condition known as Polycystic lipomembranous osteodysplasia with sclerosing leukoencephalopathy. Of note, *TREM2* plays a crucial role in microglia cells as part of the phagocytic scavenger pathway [116].

The phenomena of one gene presenting with different phenotypes is becoming more common in the context of neurological disorders and in respect to common diseases, such as PD and AD. It is important for clinicians to be familiar with these concepts in order to be able to properly counsel their patients and their family members. Also, the identification of such patients will hopefully offer more effective treatments, once available.

These new insights into the understanding of neurodegenerative diseases and, in particular, PD open new scenarios that only a few years ago were still totally obscure. Hopefully, these discoveries will be important for a real discernment of these severe conditions and for the discovery of more effective therapeutic approaches.
